# Development of a Clinical Prediction Rule to Determine Walking Independence in Older Adults With Hip Fractures

**DOI:** 10.7759/cureus.72329

**Published:** 2024-10-24

**Authors:** Taiki Iwamura, Hiroki Iwamoto, Shota Saito, Yoichi Kaizu, Shuntaro Tamura, Ren Takeda, Sota Kobayashi, Kazuhiro Miyata

**Affiliations:** 1 Department of Rehabilitation, Azumabashi Orthopedics, Tokyo, JPN; 2 Department of Rehabilitation, Hidaka Rehabilitation Hospital, Takasaki, JPN; 3 Department of Rehabilitation, Fujioka General Hospital, Fujioka, JPN; 4 Department of Physical Therapy, Ota College of Medical Technology, Ota, JPN; 5 Department of Rehabilitation, Day Care Center Specialized in Stroke Rehabilitation "With Reha", Gunma, JPN; 6 Department of Physical Therapy, Niigata University of Health and Welfare, Niigata, JPN; 7 Department of Physical Therapy, Ibaraki Prefectural University of Health Science, Ibaraki, JPN

**Keywords:** clinical decision, fall prevention, mobility, rehabilitation medicine, walking ability

## Abstract

Introduction and aim: Accurate determination of walking independence in older adults after hip fracture surgery is crucial for selecting appropriate walking strategies and providing fall prevention guidance. We developed and validated a clinical prediction rule (CPR) to determine level-surface walking independence and community walking independence in older adults with hip fractures.

Methods: In a multicenter, cross-sectional study, we investigated 289 older inpatients with hip fractures. A backward stepwise logistic regression analysis was performed to develop a CPR for determining level-surface walking independence and community walking independence. The independent variables incorporated the patients' walking and balance evaluations at discharge, including the Berg Balance Scale (BBS), the Timed Up and Go (TUG) test, and maximum walking speed (MWS), as well as age and cognitive function evaluations. We used a bootstrap internal validation for the CPR's internal validation.

Results: At their hospital discharge, 171 patients had achieved level-surface walking independence, and 51 had achieved community walking independence. As the level of walking independence decreased, patients tended to be older, with lower BBS scores and slower TUG times and walking speeds. As diagnostic factors, the level-surface walking model included age, BBS, and cognitive decline; the community walking model included BBS, TUG, and MWS. The diagnostic accuracy, represented by the area under the curve (confidence interval), was 0.88 (0.84-0.92) for the level-surface walking model and 0.81 (0.74-0.87) for the community walking model. Internal validation confirmed that the models' discrimination accuracy was good, and no model overfitting was observed.

Conclusions: We developed a moderately accurate CPR to determine walking independence in hip fracture patients. CPR can be a useful indicator for determining an individual's walking independence at present, but external validations with other samples are necessary.

## Introduction

Hip fractures occur more frequently in older adults and are often caused by falls [1,2]. In prior studies, ≥20% of patients with a hip fracture continue to have decreased walking ability and activities of daily living postoperatively compared to their preoperative levels [3-6], and 38% had fallen at least once within the prior six months [7]. It has been shown that the ability to walk at discharge affects patients' mortality at one year [8], and that outdoor walking has many broad health benefits [9]. A higher level of walking is thus beneficial for individuals who have incurred a hip fracture. However, habitual walking for individuals at high risk of falls increases the risk of falling, making it crucial to accurately assess the current walking ability of hip fracture patients and to provide appropriate guidance on activity levels and walking techniques [10].

Subjective judgments of fall risk by physicians and others may have low inter-examiner validity, and it is thus desirable to use an objective rating scale to judge patients' fall risk. Multiple factors are associated with the risk of falling [7,11], and of them, walking impairment and balance impairment are domains that most consistently predict future falls [12]. The measurement of walking speed, the Timed Up and Go (TUG) test, and the Berg Balance Scale (BBS) are effective evaluations for assessing walking and balance. These tools have been shown to help identify individuals at risk of falling [11,12]. Each of their cut-off values can be used to determine fall risk [11,13]. However, fall risk judgments based on the cut-off value of a single evaluation may not adequately take multiple factors into account. Thus, there is a need for a clinical prediction rule (CPR) that includes multiple factors to determine walking independence.

CPRs have been reported to predict the walking-ability prognosis in patients with hip fractures based on preoperative and perioperative data [3-6]. Quantitative and individualized prognostic evaluations with these CPRs can be used to customize care at an individual level. However, walking and balance evaluations that can be conducted in the perioperative period are limited and thus cannot be included as predictors. CPRs for walking and balance evaluations at a given point in time are needed to determine patients' walking independence at that point in time, but such CPRs have not been established. In addition, since fall factors differ between indoor and outdoor walking, it is necessary to determine an individual's independence in both walking on level surfaces and walking in the community, which includes stairs and uneven surfaces [7,14,15].

A clinical prediction rule (CPR) that incorporates a patient's walking and balance evaluation results, along with their age and cognitive function - both of which are linked to walking ability - would offer a more accurate assessment of the patient's independence in walking on level surfaces and in the community. If these CPRs can accurately assess walking ability just before discharge, they will facilitate the determination of appropriate walking strategies immediately after discharge at the patient's next care destination, thereby contributing to fall prevention. We conducted this study to develop and validate a CPR to determine the level of independence in walking on level surfaces and in the community for hip fracture patients, based on their current walking and balance abilities.

## Materials and methods

Study design and patients

This was a multicenter, cross-sectional study conducted at the following four hospitals in Gunma prefecture: Fujioka General Hospital, Public Nanokaichi Hospital, Hidaka Rehabilitation Hospital, and Hidaka Hospital. The inclusion criteria for patients were as follows: (i) patients diagnosed with a hip fracture between April 1, 2020, and March 31, 2022; (ii) patients admitted to a convalescent rehabilitation ward and undergone rehabilitation; and (iii) patients aged 65 years or older. The convalescent rehabilitation wards, specialized units for the subacute phase of recovery, have been part of the Japanese medical insurance system since 2000. In 2009, Japan had a total of 57,028 rehabilitation beds in these wards across the country [16]. Patients in these wards can receive up to three hours of rehabilitation therapy per day. For those suffering from hip fractures, the maximum length of stay is 90 days. The exclusion criteria were as follows: (i) severe walking impairment due to neurological or musculoskeletal diseases other than a hip fracture, (ii) missing data for all three walking and balance evaluations, (iii) refusal to participate, and (iv) difficulty walking independently prior to the injury. The reporting of the study follows the transparent reporting of a multivariable prediction model for the Individual Prognosis or Diagnosis (TRIPOD) statement [17] and the Strengthening the Reporting of Observational Studies in Epidemiology (STROBE) statement [18].

We publicized information about the study, including an opt-out option, and we ensured that patients had the opportunity to decline to participate. The study was approved by the Ethics Review Committees of Fujioka General Hospital (#270), Public Nanokaichi Hospital (#20210100), Hidaka Rehabilitation Hospital (#220402), and Hidaka Hospital (#349), and it was conducted in compliance with the Declaration of Helsinki.

Outcomes

The primary outcome of this study was the patients' walking ability at their discharge from the hospital, measured by the Functional Ambulation Categories (FAC), which assesses the testee's ability to walk steadily in a straight line on stairs and outdoors [19]. The FAC classifies walking ability into six distinct levels [19,20]. FAC=0 indicates that the individual is unable to walk. FAC=1 denotes a requirement for continuous manual contact to support body weight while walking. FAC=2 signifies that the individual requires intermittent or continuous light touch for walking. FAC=3 describes a person who can walk but depends on supervision. FAC=4 refers to individuals who can walk independently, but only on level surfaces. Finally, FAC=5 indicates full independence in walking, including the ability to navigate stairs. FAC scores have been confirmed to have high test-retest reliability and inter-rater reliability in stroke patients [20]. In the present study, each patient's FAC score was assessed by the physical therapist in charge, based on their regular walking and balance evaluation results and walking conditions during daily rehabilitation. The determination of FAC=5 considered not only walking ability but also the individual's capacity to ascend and descend stairs.

Predictors

We collected basic demographic information from patients' electronic medical records including age, gender, body mass index (BMI), fracture location and treatment, pre-admission FAC, balance function (BBS, TUG scores), maximum walking speed (MWS), and cognitive decline at discharge. From among these, we used the BBS, TUG, and MWS (which are associated with walking ability) [7,11,12], age, and cognitive decline (which are generally associated with walking ability) as predictors [3-6,12]. Cognitive decline was determined by a diagnosis of dementia before the patient's hip fracture injury or by a cognitive function test after the injury. The cognitive function tests were the Revised Hasegawa's Dementia Scale (HDS-R) or the Mini-Mental State Examination (MMSE), both of which are rated on a 30-point scale, with lower scores indicating lower cognitive function [21,22]. The cut-off values for cognitive decline were <21 points out of 30 for the HDS-R and <23 points out of 30 points for the MMSE [21,22]. Cognitive decline was scored as 1 point; no cognitive decline was scored as 0 points. Discharge walking, balance, and cognitive function evaluations were performed just before each patient's discharge by the physical therapist in charge.

The BBS test consists of the following 14 tasks: change of position (sitting to standing), standing unsupported, sitting unsupported, change of position (standing to sitting), transfers, standing with eyes closed, standing with feet together, reaching forward while standing, retrieving an object from the floor, turning trunk (feet fixed), turning 360°, stool stepping, tandem standing, and standing on one leg [23]. Each task is rated on a scale of 0-4, with 56 points being the highest possible total score [23]. Higher scores indicate greater balance ability. A period of 10-20 min is necessary to conduct all of the BBS tasks [24]. BBS scores have shown high reliability and validity in older adults [25,26].

The TUG test calculates the time it takes a patient to get up from a chair (45 cm seat height, no armrests), walk a distance of 3 m, turn around, return to the chair, and sit down. The present study's patients used their usual walking aid (if any) and were instructed to perform the TUG test as quickly as possible. High relative intratester and intertester reliabilities of the TUG test in patients with hip fractures have been reported [27].

Each patient's maximum walking speed (MWS) was measured over a 10-m distance on a flat floor with a total pathway length of 16 m. We instructed the participants to walk the 16 m distance at their fastest walking speed. The time taken to walk the 10-m distance in the middle of the 16-m long walking path was measured using a stopwatch. Patients used their usual walking aid if any. The MWS has shown high test-retest reliability in older people dwelling in the community [28].

Statistical analysis

The study utilized five independent variables as follows: three walking and balance evaluations, age, and cognitive function, all of these have been identified in previous research as being associated with walking ability. Multiple logistic regression models require at least 10 events per variable, so this study needed a minimum of 50 patients in each group [29].

We used multiple imputations to deal with missing data. We divided the patients into the following three groups based on their FAC scores at discharge: the dependent group (FAC score ≤3), the level-surface walker group (FAC=4), and the community walker group (FAC=5). The continuous variables collected are shown as the median and interquartile range (IQR).

We performed a backward stepwise logistic regression analysis to develop a CPR that can be used to determine hip fracture patients' walking independence at discharge. A significance value of 0.05 was required to enter a variable into the model. In the model to determine level-surface walking independence (the level-surface walking model), the dependent variables were defined for the level-surface walker group as 0 and the dependent group as 1. In general, in order to progress from the level of dependent walking to surface walking and then to community walking, patients who were already independent in community walking were not included in this model.

In the model to determine community walking independence (the community walking model), the dependent variables were defined for the community walker group as 0 and the level-surface walker group as 1. For both models, the independent variables were age, BBS, TUG, MWS, and cognitive decline. The relative weighting of every variable included in each model was based on each variable's β-value in the logistic regression analysis. The multicollinearity was confirmed using the variance inflation factor (VIF). We considered a VIF value of ≥10 acceptable for multicollinearity. If multicollinearity was observed, one variable was deleted. We verified the diagnostic accuracy of each CPR by obtaining the receiver operating characteristic (ROC) curves and the area under the curve (AUC) for discrimination. In addition, sensitivity and specificity were calculated based on the point where the ROC curve was closest to the upper left of the graph. An AUC value of 0.5-0.7 indicates low diagnostic accuracy; 0.7-0.9 indicates medium diagnostic accuracy, and ≥0.9 indicates high diagnostic accuracy [30].

As an internal validation of each CPR, the AUC and 95% confidence interval (CI) of the model created using the bootstrap internal validation procedure with 200 replications confirmed the over-fitting of the model. We considered the model positive if the patient was able to walk independently on a level surface or in the community. All statistical analyses were performed using the R program version 4.1.0 (Vienna, Austria: R Foundation for Statistical Computing).

## Results

The missing data were as follows: 0.3% missing values for BBS, 26.3% for TUG, 5.0% for MWS, and 6.3% for BMI. The reason for the high number of missing TUG test data is that one of the participating hospitals does not use the TUG test for routine evaluations. Table 1 summarizes the characteristics of the patients. We analyzed the data of 289 patients (229 women and 60 men), with median (IQR) age of 83.0 (76.0-88.0) years. There were 67 participants in the dependent group, 171 in the level-surface walker group, and 51 in the community walker group. The dependent group (n=67) was 66 patients with the FAC classification of 3 and one patient with the FAC of 2. The median age of the dependent group was the oldest of the three groups. In addition, 57 patients in the dependent group, 133 in the level-surface walker group, and 13 in the community walker group used some type of walking aid. As the level of walking independence fell, the BBS score tended to be lower and the TUG test result and walking speed also tended to be slower.

**Table 1:** Demographic characteristics of the participants. Data are presented as median (interquartile range) or counts. A two-tailed p-value <0.05 was considered significant. BMI: body mass index; BBS: Berg Balance Scale; TUG: Timed Up and Go Test; MWS: maximum walking speed; FAC: Functional Ambulation Categories

Variables			Overall, n=289	Walking independence at discharge		
				Dependent, n=67	Level-surface walker, n=171	Community walker, n=51
Age (years)			83.0 (76.0-88.0)	88.0 (83.0-91.5)	82.0 (75.0-87.0)	79.0 (73.0-84.5)
Genders	Males		60	10	37	13
	Females		229	57	134	38
BMI (kg/m^2^)			20.25 (18.3-22.5)	18.9 (17.2-22.4)	20.5 (18.5-22.4)	20.0 (18.6-22.7)
Evaluation date from date of injury or surgery (days)			62.0 (45.0-78.0)	69.0 (50.5-90.5)	62.0 (43.5-78.0)	52.0 (44.5-66.5)
Fracture type (inside)			155	27	99	29
Surgical form		Femoral head replacement	105	15	66	24
		Intramedullary	146	42	85	19
		Plate	17	6	6	5
		Conservative	8	3	5	0
		Others	13	1	9	3
Prefracture FAC	FAC 4		78	42	33	3
	FAC 5		211	25	138	48
Walking aid at discharge	No device		85	9	38	38
	Cane		124	17	97	10
	Walker		79	40	36	3
Walking orthosis user			6	2	3	1
BBS			48.0 (42.0-53.0)	40.0 (32.0-44.0)	49.0 (45.0-53.0)	54.0 (51.0-56.0)
TUG (s)			14.8 (11.3-22.4)	24.3 (17.5-37.2)	14.5 (11.3-20.4)	10.4 (8.8-12.4)
MWS (m/s)			0.9 (0.7-1.2)	0.6 (0.4-0.9)	0.9 (0.7-1.1)	1.2 (1.1-1.4)
Cognitive impairment	No		232	35	154	43
	Yes		57	32	17	8

As shown in Table 2, the backward stepwise logistic regression analysis extracted the following model factors. The level-surface walking model extracted patient age (odds ratio {OR}: 1.08, 95% CI: 1.02-1.15), BBS (OR: 0.87, 95% CI: 0.82-0.91), and cognitive decline (OR: 4.88 95% CI: 2.16-11.00). The community walking model extracted BBS (OR: 0.76, 95% CI: 0.66-0.87), MWS (OR: 0.05, 95% CI: 0.01-0.27), and TUG test (OR: 0.87, 95% CI: 0.80-0.94). All factors had a variance inflation factor (VIF) <10.0.

**Table 2:** Backward stepwise logistic regression analysis for level surface walking and community walking independence as dependent variables BBS: Berg Balance Scale; CI: confidence interval; MWS: maximum walking speed; OR: odds ratio; TUG: Timed Up and Go Test; VIF: variance inflation factor

Variables		β-values	OR	95% CI	VIF	Sensitivity	Specificity	AUC	AUC 95% CI
Level-surface walk model	Intercept	-1.69	-	-	-	0.90	0.54	0.88	0.84-0.92
	Age (years)	0.08	1.08	1.02-1.15	1.01				
	BBS	-0.15	0.87	0.82-0.91	1.74				
	Cognitive decline	1.59	4.88	2.16-11.00	1.01				
Community walk model	Intercept	20.39	-	-	-	0.25	0.93	0.81	0.74-0.87
	BBS	-0.27	0.76	0.66-0.87	2.44				
	TUG (s)	-0.14	0.87	0.80-0.94	3.77				
	MWS (m/s)	-2.96	0.05	0.01-0.27	2.29				

**Figure 1:**
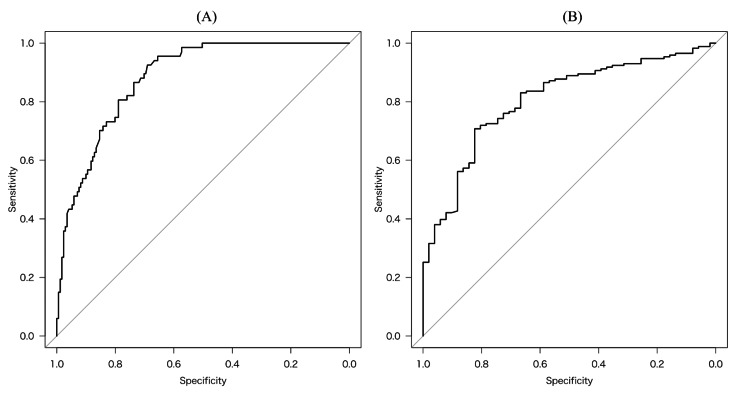
ROC curves for CPR scores corresponding to each degree of of walking independence at discharge. The ROC curves show the prediction accuracy for (A) the level-surface walk model and (B) community walk model. ROC: receiver operating characteristic; CPR: clinical prediction rule

The following regression equations were calculated from the β-values of each model. For the level-surface walking model, dependent walker: p>0.5, level-surface walker: p<0.5 = 1/{1+exp - (-1.685 + 0.079 × age - 0.145 × BBS + 1.586 × cognitive decline)}. For the community walking model, level-surface walker: p>0.5, community walker: p<0.5 = 1/{1+exp - (20.39 - 0.273 × BBS - 0.144 × TUG - 2.960 × MWS)}. The level-surface walking model showed 0.90 sensitivity and 0.54 specificity values, and the AUC (95% CI) was 0.88 (0.83-0.92). The community walking model showed 0.25 sensitivity and 0.93 specificity, and the AUC (95% CI) was 0.81 (0.74-0.87) (Table 2 and Figures 1A, 1B).

The internal validation using the bootstrap method resulted in a mean AUC of 0.87 and a slope of 0.96 for the level-surface walking model and a mean AUC of 0.79 and a slope of 0.93 for the community walking model. The discrimination accuracy was thus moderate, and no model overfitting was observed.

## Discussion

This study aimed to develop and validate CPR to determine walking independence among hospitalized patients with hip fractures based on the patient's ability to walk and his or her balance at discharge. The results showed that the patient's age, BBS score, and cognitive decline were included in the level-surface walking model, and the BBS score, TUG test score, and MWS were included in the community walking model. Both models showed moderate diagnostic accuracy. Our finding that multiple factors were extracted in both models supports earlier reports recommending a multifactorial assessment with regard to walking ability [11,12].

The discriminability of the model was AUC 0.88 for the level-surface walking model and 0.81 for the community walking model. The AUCs in earlier studies of walking-ability CPRs for hip fracture patients were 0.68-0.85, indicating that the CPR that we identified in the present study has slightly better accuracy compared to the previously reported CPRs [3,5-8]. The new CPR was thus demonstrated to be a useful indicator for determining a hip fracture patient's present walking ability. Our internal validation using the bootstrap internal validation procedure with 200 replications showed that the AUC values for both models were similar to the original data, with slopes ranging from 0.9 to 1.0 for both. This indicates that the model is not overfitting and is sufficiently reproducible.

The sensitivity and specificity values of the level-surface walking model were 0.9 and 0.54, respectively, and those of the community walking model were 0.25 and 0.93, respectively. The level-surface walking model thus has higher sensitivity than specificity and may help to identify patients whose level-surface walking independence may be at risk. The BBS included as a predictor in this model is reported to have 0.41 sensitivity and 0.88 specificity at a cut-off value that can be used to identify community-dwelling older people at risk of falling [11]. It may thus be possible to identify patients with level-surface walking independence who are still at risk of falling by adding age and cognitive function with higher accuracy compared to an evaluation based on the BBS alone. The community walking model thus has high specificity and may help identify patients for whom community walking independence is possible. However, the model's sensitivity is 0.25, which is low and may lead to misclassification, and thus care should be taken in its use.

In the present patients' walking and balance evaluation, the BBS was included in both the level-surface walking and community walking models, whereas the TUG test and MWS were included only in the community walking model. Our results suggest that the balance assessment by the BBS is more useful than the walking speed or TUG score in determining indoor walking independence. The TUG test and MWS require walking as quickly as possible, whereas the BBS consists of 14 tasks to evaluate balancing skills [23]. Patients who are aiming for indoor walking independence may therefore need to focus on gaining balance skills first. The fact that only walking and balance evaluations were included in the present community walking model suggests that compared to indoor walking, outdoor walking requires higher walking balance ability.

The community walking model showed a regression equation in which the faster the patient's TUG test result is, the more dependent the patient becomes. The TUG test includes the BBS sub-item "positional change (sitting to standing)" and the walking speed factor [28]. The inclusion of the BBS score and the MWS (which have elements that are similar to those of the TUG test) as factors in the model may have led to this result due to the lower influence of the TUG result in walking independence. The β-value of the TUG test in the regression equation was -0.14, and the clinical impact is expected to be small.

This study has several limitations. The first is that the CPRs obtained in this study cannot be used to predict walking ability. These CPRs assess patients' walking independence at the time they are used, so it's important to use them cautiously, as they do not predict walking ability over time. The second limitation is that the total number of patients was small and the number of patients in each group was not balanced. The small number of community walkers in particular (n=51) may have created a model that ignored a correct classification of community walkers. It is therefore necessary to further increase the number of patients and consider aspects of statistical analyses such as oversampling. The third limitation is that we did not take into account other walking and balance evaluations. Self-selected walking speed and the averaged five-times sit-to-stand time and other parameters have been reported as factors associated with walking ability [11]. It may be possible to create a more accurate model by increasing the number of subjects and analyzing more factors. In addition, several evaluation methods are not standardized. This applies to cognitive function evaluations and the influence of walking aids in walking evaluations. The walking evaluation conducted in the present study did not include the consideration of walking aids. It is necessary to take into account the possibility that some of the monitored patients were able to walk independently with the use of walking aids, although walking alone was monitored. Lastly, no external validation was conducted in this study. Our results should be validated in an external cohort.

## Conclusions

We developed a CPR to determine independence in walking on level surfaces and independence in community walking for patients with hip fractures. We used patient age, the BBS score, and the cognitive function result to examine level-surface walking independence and the BBS, TUG test, and MWS for community walking independence to predict walking independence. Each of the developed models consists of three simple assessment items, which we believe are clinically applicable.
